# Society Meetings

**Published:** 1911-05

**Authors:** 


### SOCIETY MEETINGS

---

The Medical Society of the State of New York held its one hundred and fourth annual meeting at Albany, Tuesday and Wednesday, April 18, and 19, 1911, under the presidency of Dr. Charles Stover, of Amsterdam. The annual banquet was held at The Ten Eyck on the evening of April 19.

Buffalo physicians who were represented at the meeting by the reading of papers or participation in discussions were: Charles G. Stockton, E. R. McGuire, Allen A. Jones, A. L. Benedict, F. C. Busch, Henry Adsit, Lucien Howe, and Harvey R. Gaylord. The provisional program was as follows:

Report of chylous cyst of mesentery and statistics of literature, A. L. Benedict.

Studies on a local hematological factor in the causation of uterine hemorrhage, A. Sturmdorf.

Operation for adherent retrodisplaced uteri by shortening the round ligaments through the inguinal canal after abdominal section through the transverse incision, Le Roy Broun.

Concerning the treatment of bladder tumors with the high frequency current of Oudin, Edwin Beer.

Diagnosis and surgical indications of duodenal ulcer, James T. Pilcher.

The new thoracic department of the German Hospital of New York, Willy Meyer.

Taking the cure at Carlsbad, Harry G. Watson.

The bladder and the prostate, John F. W. Whitbeck.

Symposium on Salvarsan, arranged by James Winfield.

The relationship of tabes to general paresis—Are they the same disease differing only in the situation of the lesion? Edward D. Fisher.

Paralysis of the upper extremity due to nerve injury, Nathan Jacobson.

The nervous woman, Edward B. Angell.

Papers with lantern demonstration:

Vaccination in the Army, Col. John Van Hoff, Medical Corps, Fort Jay, New York.

The clinical observations of cardiac arrythmias from the modern standpoint, Walter B. James.

Diagnosis of some common tropical infections, S. M. Shook, Passed Assistant Surgeon, U. S. N.

Fractures of the tarsal bones, with radiograms, Fred H. Albee.

Radiographic diagnosis of lesions of the gastrointestinal tract with lantern slide demonstration; and cinematographic demonstration of the peristalsis in normal and pathological cases, Lewis G. Cole.

Hypernephromata; the effect of their extracts upon blood pressure, F. C. Busch.

Arteriosclerosis, Louis Faugeres Bishop.

The significance and therapy of high blood pressure, J. F. Rooney.

The hydrotherapeutic treatment of chronic disease of the heart, John M. Swan.

Some orthodiascopic observations with reference to the size of the heart and its relation to certain general systemic affections, Isaac Adler.

The operative treatment for fractures, M. M. Lucid.

Olive oil in post-operative nausea, Clarence B. Hyde.

Cerebral compression, Edgar R. McGuire.

Developmental defects of the abdominal viscera with their surgical significance, William F. Campbell.

One hundred and seventeen consecutive operations for exophthalmic goiter without fatality, Martin Tinker, Cornell University.

Deductions from clinical experience in the use of a polyvalent bacterial vaccine, J. M. Van Cott.

Mutual helpfulness in the conservation of public health, William A. Howe.

The prevention of insanity, Albert Warren Ferris.

Ausculation of the cough—its importance, A. H. Garvin.

Malignant tumors of the kidney, Henry Adsit.

The diagnosis of diseases of the kidney, Paul M. Pilcher.

Exophthalmos, a common symptom of Bright's disease, H. C. Gordinier.

The method for preventing gonorrheal ophthalmia, Lucien Howe.

The precancerous stage, Parker Syms.

Cancer immunity in its present status, Harvey R. Gaylord.

The etiology, pathology, symptoms, diagnosis and treatment of intestinal tuberculosis, Samuel G. Gant.

Gunshot wounds of the abdomen, Edgar A. Vander Veer.

Chronic appendicitis, a critical study of post-operative end results, E. MacD. Stanton.

A new method of end to end anastomosis, A. L. Soresi.

Exhibition of pathological material by H. Oertel, of Russell Sage Foundation.

Pathological exhibit from Syracuse University, arranged by Professor Steensland.

The election of officers for the ensuing year resulted as follows: President, Wendell C. Phillips, New York; vice-presidents, Peter W. Van Peyma, Buffalo, William P. Campbell, Brooklyn, C. R. Hervey, Oswego; secretary, W. R. Townsend, New York, (reelected); treasurer, Alexander Lambert, New York.

The society voted to meet in Albany next year, declining the invitation from Rochester.

---

The Buffalo Academy of Medicine held meetings during March and April as follows:

*Section of Obstetrics and Gynecology.*—Tuesday evening, March 28. Program: The treatment of post-operative peritonitis with report of cases, Hunter Robb.

*Section of Surgery.*—Tuseday evening, April 4. Program: Cancer of the stomach, Herbert A. Bruce, Toronto.

*Section of Medicine.*—Tuesday evening, April 11. Program: A diagnostic point in the timing of heart murmurs, Eli H. Long; The therapy of heart-block, Walter A. Bastedo, New York; Report of a case of heart-block, Nelson G. Russell.

*Section of Obstetrics and Gynecology.*—Tuesday evening, April 25. Program: Toxemias of the latter part of pregnancy, F. C. Goldsborough; Differential diagnosis of intra-uterine pregnancy, Ludwig Schroeter.

---

THE Rochester Academy of Medicine held meetings during April as follows:

*Section of Medicine.*—Wednesday evening, April 5. Program: Report of cases, J. R. Culkin, N. W. Soble, C. E. Darrow and C. D. Young.

*Section of Surgery.*—Wednesday evening, April 12. Program Labyrinthine Suppuration, Leonard W. Jones.

*Section of Obstetrics.*—Wednesday evening, April 19. Program: Uterine retrodisplacement—its surgical treatment, Charles R. Barber; Discussion of its medical treatment, Charles E. Darrow.

*Section of Public Health.*—Wednesday evening, April 26. Program: Some cases treated by vaccines, Charles O. Boswell.

---

THE Buffalo Physical Education Society, of which Dr. James Wright Putnam is president, was addressed on Monday evening, April 10, at the Perkin's Memorial Hall, Central Y. M. C. A. Building, by Dr. William E. Watt of Chicago, on the topic "Pure Air for Public Buildings." Dr. Watt was formerly principal of the now famous Graham School of Chicago, where he developed his ideas in relation to the humidifying of pure air, thereby increasing the vitality and efficiency of his pupils. By analogy he holds that the same principle should apply to all dwellings—our private homes as well as public buildings. In the audience were officials from the public schools, teachers, janitors, physicians, and others interested in public health.

---

THE Oregon State Board of Medical Examiners, the Louisiana State Board of Medical Examiners (regular), Dr. R. S. Copeland, New York, Dr. James H. McDonald, Pittsburg, Dr. F. F. Lawrence, Columbus, and Dr. C. M. Hazen, Bon Air, Va., were admitted to membership in the National Confederation of State Medical Examining and Licensing Boards at its recent annual meeting held at Chicago.

---

THE American Medical Association will hold its sixty-second annual meeting at Los Angeles, Cal., June 27-30, 1911, under the presidency of Dr. William H. Welch, of Baltimore. Dr. J. B. Murphy, of Chicago, is the president-elect. Dr. Allen A. Jones, of Buffalo, is the chairman of the Section of the Practice of Medicine.

